# Children’s Individual Differences in the Responses to a New Method for Physical Education

**DOI:** 10.3390/sports12120328

**Published:** 2024-11-29

**Authors:** Sara Pereira, Carla Santos, José Maia, Olga Vasconcelos, Eduardo Guimarães, Rui Garganta, Cláudio Farias, Tiago V. Barreira, Go Tani, Peter T. Katzmarzyk, Fernando Garbeloto

**Affiliations:** 1 Centre of Research, Education, Innovation and Intervention in Sport (CIFI2D), Faculty of Sport, University of Porto, 4200-450 Porto, Portugal; carlass@fade.up.pt (C.S.); jmaia@fade.up.pt (J.M.); olgav@fade.up.pt (O.V.); eguimaraes@fade.up.pt (E.G.); ruigarg@fade.up.pt (R.G.); claudiofarias@fade.up.pt (C.F.); fegarbeloto@gmail.com (F.G.); 2 Research Center in Sport, Physical Education, and Exercise and Health (CIDEFES), Faculty of Physical Education and Sports, Lusófona University, 1749-024 Lisboa, Portugal; 3 Department of Exercise Science, Syracuse University, Syracuse, NY 13244, USA; tvbarrei@syr.edu; 4 Motor Behavior Laboratory, School of Physical Education and Sports, University of São Paulo, São Paulo 05508-070, Brazil; gotani@usp.br; 5 Pennington Biomedical Research Center, Baton Rouge, LA 70808, USA; peter.katzmarzyk@pbrc.edu

**Keywords:** children, fundamental movement skills, assessment, individual differences

## Abstract

Children’s fundamental movement skills (FMS) require planned and guided interventions to develop appropriately. We investigated the effect of a novel Physical Education (PE) method to develop children’s object control, locomotor skills, and motor competence. Further, we examined children’s trainability, i.e., their differential responses to the new method, and identified low and high responders to the intervention. The study lasted three months and included six to seven-year-old children in two groups: control (the current, official PE program; *n* = 38) and experimental (the new method; *n* = 52). Twelve FMS [object control (OC), locomotor (LO)] were reliably assessed using the Meu Educativo^®^ app. Using a mixed-effects model, results showed that the experimental group experienced greater changes (*p* < 0.05) than the control group in OC and LO. Positive individual changes were more frequent with the new method, but children showed a similar pattern in their interindividual variability in both methods. There was a greater reduction in the number of children with lower proficiency in the experimental group. In sum, the new PE method proved superior to the current, official one. Individual responses to the new method showed considerable variation, highlighting the need for personalization in teaching strategies and necessary support for children with lower proficiency levels, ensuring that no child is left behind in their motor development process.

## 1. Introduction

Children’s fundamental movement skills (FMS) are widely acknowledged as essential building blocks of more refined and culturally related skills, the epitome of which are sports skills [1,2,3]. It is now recognized that children’s FMS proficiency levels are positively related to their physical activity and health trajectories, including a lower likelihood of being overweight or obese [4,5,6]. Furthermore, it has been shown that proficiency in FMS during childhood positively affects physical fitness during adolescence [7]. We have recently reported that children with higher FMS levels were more physically active than their peers with lower FMS levels, both on weekdays and weekends [8]. We have also found that children with obesity, those who are sedentary, and those not meeting the World Health Organization’s guidelines for moderate-to-vigorous physical activity [9] were less likely to have a higher FMS score [10].

In structural terms, the FMS repertoire is usually clustered into three groups [11,12,13]: object control (e.g., overarm throw, catching, striking, kicking, rolling), locomotor (e.g., running, galloping, sliding, jumping), and stability (e.g., bending, twisting). The proficiency with which a given child integrates these skills in various settings displays her/his motor competence. Moreover, it is generally acknowledged that children’s FMS are expected to be mastered, i.e., revealing a mature movement pattern, by the end of their primary school years (eight to ten years of age) [11,14]. For this to happen, these skills must be systematically taught and practiced in formal and well-designed educational programs, specifically in physical education (PE) classes [15]. Notwithstanding PE classes’ key roles during the first school years, there is evidence that without appropriate methodologies, these classes may not improve FMS performance, especially in children with low performance [16,17,18].

Research on FMS development is available for preschool-aged children. For example, a three-year intervention study showed that movement skill competence increased proficiency in object control skills [19]. It has also been reported that a structured skill-based eight-week intervention program positively affected preschoolers’ FMS [20]. Similar trends have been shown to exist in primary school children’s FMS development, as described by Bryant, et al. [21] with a six-week physical activity intervention, and in locomotion skills and physical competence with a 12-week home, school, and community-based physical literacy intervention [22]. A systematic review by Han, et al. [23] showed that children with overweight/obesity tended to improve their FMS proficiency levels with proper exercise/physical activity interventions within their PE curriculum, and a similar pattern occurred in children with impaired motor skills [13]. Notwithstanding the relevance of these findings, it should be acknowledged that the main interest of these studies was in main effects, i.e., mean changes from pre- to post-intervention, and never addressed the issue of children’s differential responses to the interventions expected to exist beyond measurement error.

It is well known that the design and analysis of observational and experimental research studies in developmental sciences, especially in Motor Development, are far more complex than meets the eye [24,25,26] and should probably explore additional analyses beyond using the analysis of variance to test for differences among means. Two of the most relevant issues regarding experimental studies are the analysis and interpretation of interindividual differences in response to interventions [27,28] and trainability [29]. Most of the research tackling these issues started with the HERITAGE Family Study (Bouchard, et al. [30], and for a recent overview, see [31]). There is a wealth of research on adults’ response heterogeneity in resistance training [32,33,34,35], and a critical review on interindividual responses in maximal oxygen uptake with its links to precision medicine is available [36]. These and the HERITAGE reports showed how important it was to investigate the extent to which subjects respond very differently to interventions, and this has not been addressed in FMS research with children.

Haywood KM and Getchtel N [3] suggested that individual differences in children’s growth and motor development are pervasive. Yet, to our knowledge, the issues of interindividual differences in response to educational interventions and trainability are seldom addressed in research on children’s FMS development within the school context. In our view, these are relevant issues when designing, implementing, and assessing “old” and new methods for PE teaching targeting FMS mastery, especially for children who may be putatively considered low responders or showing difficulties in their performance levels. This is important for PE teachers, especially when planning classes, so that no child should be left behind.

It is also important to recognize that available studies typically focus on reporting average outcomes from intervention programs, thereby leaving gaps in our understanding of how they specifically impacted children with low FMS proficiency levels. It is important to emphasize that these children are particularly vulnerable in developing more advanced skills and complying with physical activity recommendations [5,37]. One also has to acknowledge that while intervention studies have tested programs of varying durations, most involve children with at least two weekly PE classes. Yet, the reality of PE curricula varies globally, with some countries offering four classes per week, whereas others provide only one [38]. Therefore, assessing an intervention program’s impacts while considering these factors is vital for understanding their overall relevance.

In this article, we will tackle these issues using data from an intervention study with a new method for PE based on FMS following Clark´s [15] serious concern that FMS does not evolve without well-designed and carefully assessed interventions. The new method is strongly based on a developmental approach and distinguishes itself from traditional methods by integrating the physical, motor, psychological, and social domains at each developmental stage. Its internal structure and defined conditions (see Section 2.2) offer PE teachers a practical “building block” that supports motor and physical development while also incorporating historical, cultural, and social content.

Hence, we will first address children’s changes in object control, locomotor skills, and motor competence. Second, we will estimate children’s trainability, i.e., their interindividual differences in response to the new method beyond random variation. Third, and as a complementary examination of the efficacy of the new method, we will probe into children’s changes in their distinct FMS categorical proficiency levels. The following hypotheses will be tested: (1) the new method will be more effective than the traditional one in developing children’s object control and locomotor skills as well as in motor competence, even when adjusted for their age, sex, and number of physical education classes; (2) children’s trainability will show individual differences with more positive results in the new method; (3) children with low levels of FMS proficiency will benefit from the intervention program in increasing their motor skills performance.

## 2. Materials and Methods

### 2.1. Background of the Study

This study is a part of the RUSH research project (Return-to-school after COVID-19: The key roles of family, school, and communities on children’s growth and motor development). In brief, RUSH’s main goals were to investigate primary school children’s physical growth, motor development, motor performance, health behaviors, and cognitive aspects (memory and attention). Furthermore, following Bronfenbrenner’s bioecological theory [39], data were also gathered on their school contexts, families, and living environments in Portugal—the northern mainland (Matosinhos area) and in the autonomous region of the Azores.

The RUSH research project was planned to be implemented in three parts. Part one comprised a cross-sectional observational study with children aged 6 to 10 years. Part two was a short longitudinal observational study investigating children’s FMS development over 12 months. Part three contained two short intervention studies based on a new method for PE grounded on motor competence development. The present study provides results from the first of these two intervention studies.

**Figure 1:**
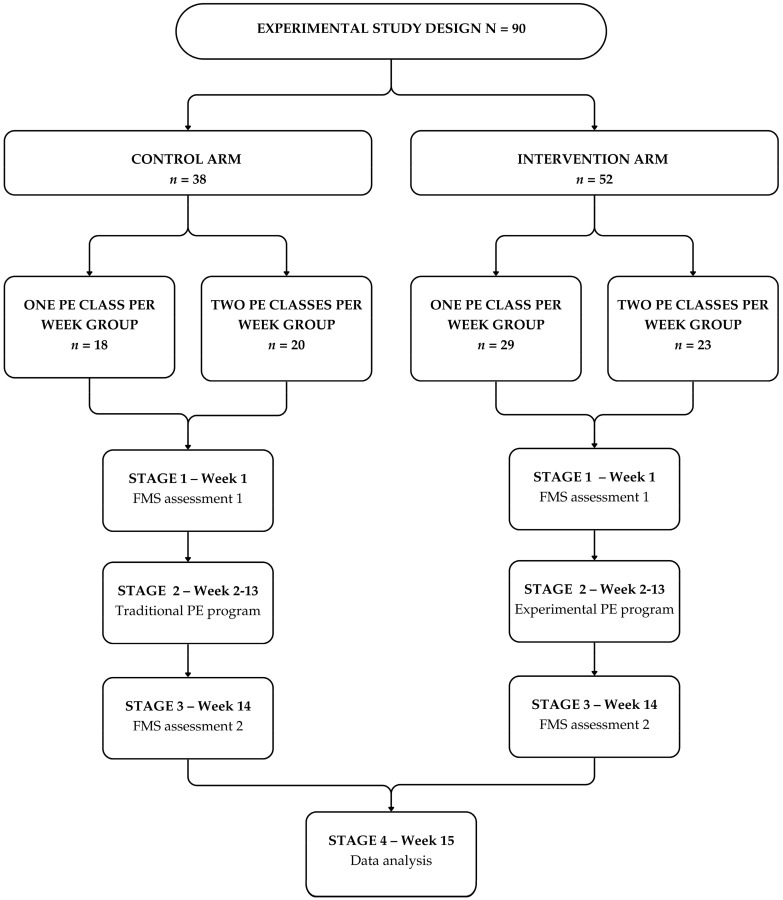
Graphical display of study design.

Parents/legal guardians provided signed informed consent for children to participate in the study. Furthermore, children could stop participating in the study at any time. The RUSH research project was approved by the school coordinators and the Ethics Committee of the Faculty of Sports, University of Porto (CEFADE 13 2022). (Figure 1).

### 2.2. The New Method

**Figure 2:**
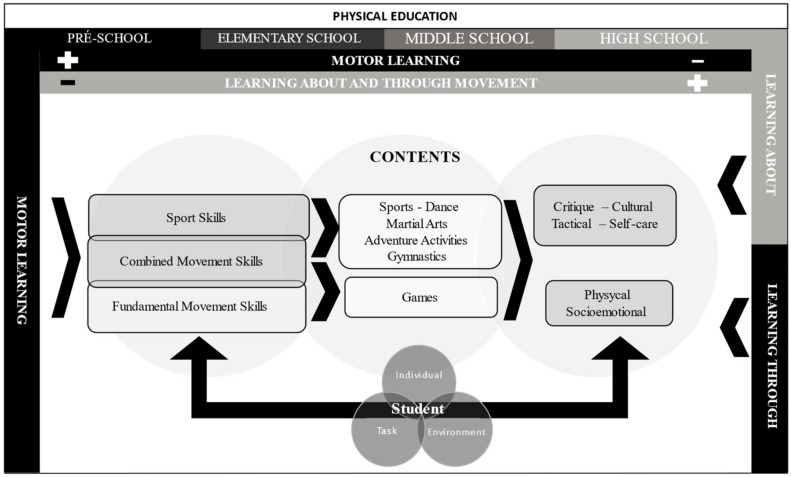
Graphical display of the essence of the method used in the experimental study. Positive (+) and negative (−) signs show the degree of emphasis in teaching and learning.

The new method, first described by Tani G, et al. [40], was recently updated by Garbeloto, et al. [41], and its essence is graphically presented in Figure 2. Its main roots are grounded in three learning possibilities: learning the movement (on the top and right), learning about movement, and learning through movement (on the top and left). Since the present paper only focuses on the first two years of primary school, the positive sign in the top bar indicates that PE teachers should emphasize the process of teaching and learning movement. It is essential to acknowledge that the negative sign in Figure 2 does not necessarily indicate the absence of learning. Learning about and through the movement was also addressed but to a minor degree, namely knowledge about movement, self-care, and cultural aspects.

The new method also considers the Newell [42] constraints model centered around three vectors: (i) children’s individual characteristics (e.g., height, weight), (ii) environment factors (e.g., available physical spaces), and (iii) task-related characteristics (e.g., skill requirements). These factors naturally shape how children develop proficiency in motor skill learning and their understanding of other domains, namely cultural and tactical aspects. Additionally, we relied on the constraints model to assist PE teachers in designing classes for children with special needs (e.g., Autism, Down Syndrome). The method also contains the relevant input from psychology, namely autonomy in the learning process [43,44]. The new method allows the teacher the freedom to determine the content of their lessons, yet certain rules must be followed: (1) lesson planning should present a sequence of content to foster the development of the same FMS. For example, the first four classes should consist of content promoting the motor skill running; (2) in addition to the rule of sequence, the PE class contents should promote diversity in the performance of the same skill (e.g., running at different speeds or directions) and increase their complexity (e.g., combining running with other skills); (3) teacher guidance on improving movement execution should occur during classes, especially for children with more significant difficulties (e.g., working with cues in learning movement); (4) whenever possible, a critical, cultural, and/or tactical component should be included, as well as another important component—promoting self-care regarding the content being practiced in class; (5) individual and environmental constraints regarding child development in different phases should also be considered when elaborating the class contents. This rule is of utmost importance for considering inclusive students (for example, students with physical disabilities); (6) the structure of the classes should promote autonomy in learning.

### 2.3. The Implementation of the New Method

In Matosinhos, irrespective of the official PE curriculum issued by the Portuguese Ministry of Education, the city hall educational department directs, organizes, and coordinates all PE teaching in primary schools. This department also hires all PE teachers according to a selection process based on the teachers’ curriculum vitae, especially their experience in teaching primary school children.

The decision to only consider the first two years of primary school was based on how the official PE curriculum was designed, i.e., in these years, all physical activities are clustered around popular games and manipulative tasks (object control) taught by graduate and certified PE teachers. The other two school years focus on initiating sports (e.g., swimming, gymnastics, ball games, roller skating, and dance).

After consulting with the city hall education department, school directors, and class teachers, we designed an experimental study with two arms based on a convenience sample from all schools given their educational constraints—a control arm (followed by three schools) and an experimental arm (followed by four schools). The control group comprised children following the traditional/official PE program issued by the Portuguese Ministry of Education, whereas the experimental group followed the new method. Furthermore, according to the number of PE classes per week and again given the school´s constraints, each arm had children with one and two PE classes per week. In the control group, we had children with one hour of PE per week, totaling 12 h of teaching and learning, and two hours per week, totaling 24 h of teaching and learning. The same pattern occurred in the experimental arm. It is important to stress that children attending these schools have similar socio-economic backgrounds and physical activities during the day. Please keep in mind that these are six to seven-year-old children on the verge of unfolding their FMS.

We had boys and girls in all classes, and no differentiation in children’s gender was made when planning and implementing all PE classes in the control and the experimental arms. Children in the control and experimental groups were part of their regular PE classes, with an average of 20 students, and not all participated in the study. Yet only those with signed consent were formally assessed. This ecological setting of the study is far different from what is usually seen in a classic experimental study [13].

One of the research team members previously trained PE teachers in all aspects of the new method. Additionally, two research team members supervised its application by PE teachers during their regular classes. The intervention lasted for three consecutive months, from March to the end of May.

### 2.4. Fundamental Movement Skills Assessment

In the RUSH research project, all FMS assessments were performed with the digital platform Meu Educativo^®^, and a complete description of its overall design, operational structure, validity, and reliability can be found in Garbeloto, et al. [45]. In RUSH, twelve FMS were considered: five object control skills (underhand roll, overhand throw, catch, kick, and stationary dribble), six locomotion skills (slide, run, horizontal jump, hop, gallop, and leap), and one stability skill (single leg position). These skills were assessed with a process-oriented approach and rated on an ordinal scale—from 1 to 3 expressing increasing levels of FMS proficiency. In operational terms, each child executed each skill at least twice; if there was any ambiguity in the child’s performance, the rater asked the child to do one or two more repetitions. Based on the last valid attempt, the child was categorized into three distinct proficiency levels using an ordinal scale (from 1 to 3): explorer climber (beginner performance: score of 1), adventurous climber (intermediate performance: score of 2), and wizard climber (advanced performance: score of 3).

The intra-rater reliability (Cohen´s kappa) ranged between 0.46 and 0.94 for object control skills and between 0.69 and 0.95 for locomotor and stability skills. For the object control skills, the final rating was the sum of the five skills and varied from 5 to 15; in locomotor, we summed the six skills plus the stability skill, and the results ranged from 6 to 18. The global rating expressing motor competence (five object control skills + six locomotion skills + one stability skill) varied from 12 to 36.

### 2.5. Sample

Based on prior arrangements with the education department of the city hall, within each school and primary school year (only the first two), children were randomly sampled from an anonymous list of all children enrolled in these school years. Children with no consent form signed by parents/legal guardians were not part of the intervention study (control and experimental arms). Further, in these classes, we did not find any disabled children.

It is important to report that children’s school environmental conditions were relatively similar. Similarly, all teachers involved in the study had similar experiences in teaching children PE, and all were graduates of Sport Sciences.

As previously mentioned, the design had two arms—a control arm and an experimental arm. In each of these arms and, based on their school educational settings and curriculum organization, children had one or two physical education classes per week, and all classes were mixed with boys and girls. In the control arm, 18 children had one physical education per week, whereas 20 had two classes. On the experimental arm, 29 had one class per week, and 23 had two classes. These discrepancies occurred because the number of informed signed consent forms differed in all classes and arms.

### 2.6. Data Analysis

We used the linear mixed-effects model with schools as the random cluster to address aim one. To test for the efficacy of the experimental program versus the control group, we added an interaction term (condition-by-time) as well as age and sex as covariates. Further, we used the margins command to predict the means of both groups adjusted for all covariates in the model. STATA 18 software [46] was used in these analyses. Additionally, and based on previous suggestions from research on individual differences [33,34], we also calculated individual changes, irrespective of gender and the number of PE classes, within each group (control and experimental) and expressed them in percentage terms according to the formula: $Delta\left(\%\right)=\left(\frac{{OC}_{M2}-{OC}_{M1}}{{OC}_{M1}}\right)100$, where OC_M2_ corresponds to results obtained at the end of the study in object control skills, and OC_M1_ are results at the beginning of the study. Similar calculations were made for locomotor skills and motor competence. Then, based on an approach introduced by Bouchard and Rankinen [47], we plotted the individual variation in pre-post differences against the cumulative number of observations after sorting from the least to the greatest change within children in each group in all skills.

To address aim two, using the raw scores of object control, locomotion, and motor competence, children´s trainability was estimated based on the following formula as previously advocated [27,28]: ${SD}_{true}=\sqrt{{SD}_{{Exp}^{2}}-{SD}_{{Cont}^{2}}},$ where ${SD}_{true}$ is the true interindividual variability in responses in the new method, for example, in the experimental group adjusted for the influence of random biological variation and measurement error; ${SD}_{{Exp}^{2}}$ is the observed standard deviation (interindividual variability) of the change scores in the experimental group (this value is squared), and ${SD}_{{Cont}^{2}}$ is the observed standard deviation (interindividual variability) of the change scores in the control group (this value is squared). Furthermore, following, the standard error of the ${SD}_{true}$ was calculated, and the corresponding 95% confidence interval. Additionally, as suggested by Dankel and Loenneke [48], a visual display (Box plot with jittering points) was used to show differential responders.

We finally described FM changes from the exploratory level within the control and experimental groups to address aim three.

## 3. Results

Table 1 shows the mixed-effects model results for object control, locomotor skills, and motor competence. The most important result is the statistically significant (*p* < 0.05) interaction condition (experimental versus control) by time adjusted for the other covariates in the model. In sum, it shows that the experimental program was more effective than the traditional one in increasing children´s fundamental movement skills.

Following the results for each model, we derived the adjusted means and their standard errors in both groups at the baseline and end of the study (see Table 2). The results showed that the means were practically the same in the traditional program (control group).

**Figure 3:**
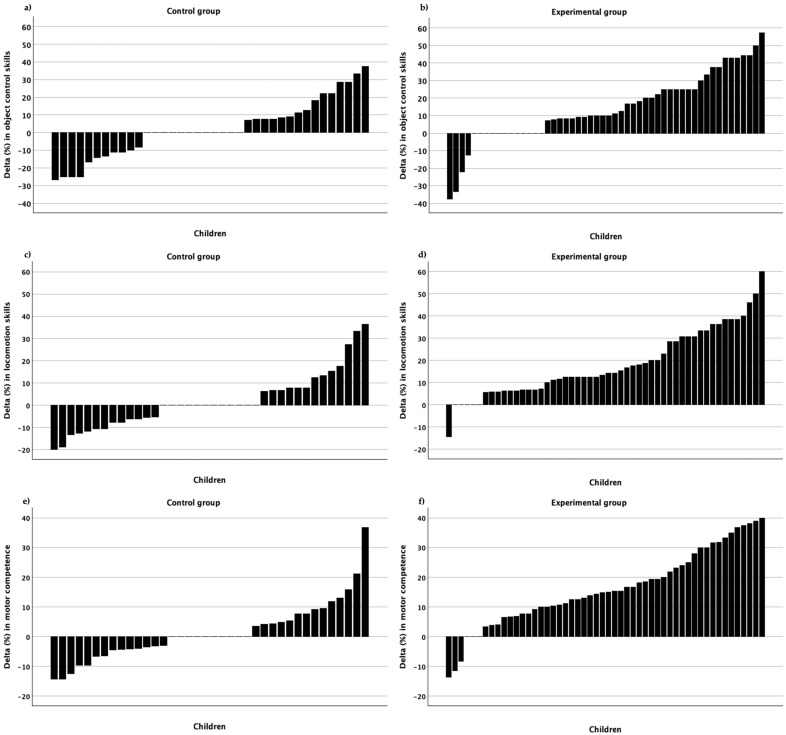
Individual changes (Delta in %) in object control skills, locomotion skills, and motor competence in the control group (**left** panel) and the experimental group (**right** panel); (**a**) Object control skills in the control group; (**b**) Object control skills in the experimental group; (**c**) locomotion skills in the control group; (**d**) locomotion skills in the experimental group; (**e**) motor competence in the control group; (**f**) motor competence in the experimental group.

Figure 3 shows variation in children’s responses in both the control and experimental groups irrespective of gender and the number of PE classes. The upper part of this figure relates to object control skills in both groups—some experienced a negative change (control group, *n* = 11; experimental group, *n* = 4), some with no change (the Delta value was zero: control group, *n* = 12; experimental group, *n* = 12), and some changed positively (the majority). In locomotion skills changes (middle part of the figure), a similar picture is seen—some children changed negatively (control group, *n* = 13; experimental group, *n* = 1), some did not change (control group, *n* = 12; experimental group, *n* = 5), and some responded positively (the majority). Finally, in motor competence, there were also children with a negative change (control group, *n* = 14; experimental group, *n* = 3), some with no change (control group, *n* = 10; experimental group, *n* = 3), and there were those who changed positively (the majority).

**Figure 4:**
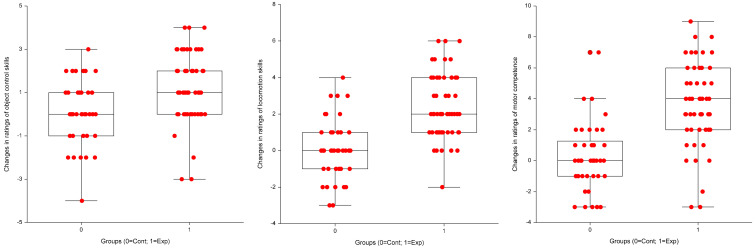
Box plot with jittering points showing children’s interindividual responses variability in object control skills, locomotion skills, and motor competence (control group labeled 0, and experimental group labeled 1).

Aim two results addressing the issue of children’s trainability using raw data, i.e., their true interindividual variability beyond random variation and measurement error in the intervention group were as follows: in object control skills, =0.55 (95%CI = −0.82 to 1.93); in locomotion skills, ${SD}_{true}=0$.62 (95%CI = −1.16 to 2.39), and in motor competence ${SD}_{true}=1$.62 (95%CI = −1.90 to 5.17). Furthermore, Figure 3 illustrates these variabilities across object control skills, locomotion skills, and motor competence (for a more detailed representation across groups conditional on the number of physical education classes, see Figure 4). Since all 95% CI contains zero, the true interindividual variability is equal in both groups—control and experimental. Remember that the control group was also undergoing regular physical education classes, and their responses are expected to also vary beyond measurement error.

**Figure 5:**
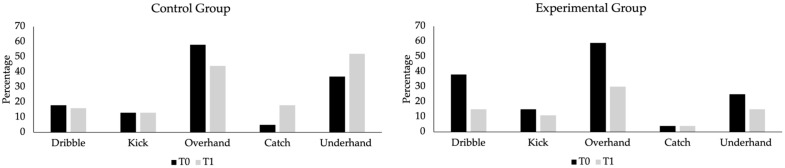
Percentage of explorer-level children in object control skills for the control and experimental groups before (T0) and after (T1) the intervention program.

**Figure 6:**
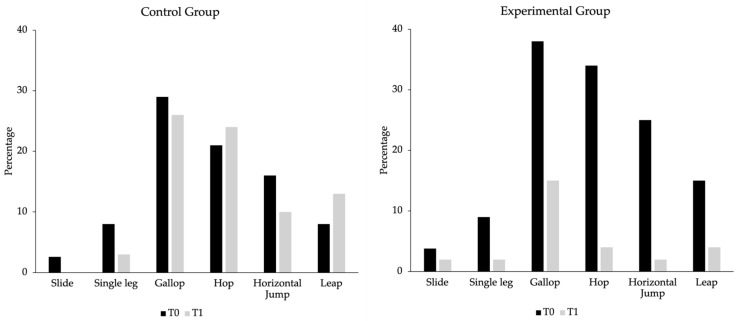
Percentage of explorer-level children in locomotor skills for the control and experimental groups before (T0) and after (T1) the intervention program.

Regarding aim three, Figure 5 and Figure 6 display the percentage of children that changed from the beginner level (explorer) to adventurous and/or wizard levels for each of the 12 FMS, except for running, where no child was classified as an explorer. Figure 5 illustrates these changes in object control skills. In the experimental group (right side of the figure), there was a decrease in the percentage of children classified at the explorer level in all tasks after the intervention program (ranging from 11% in the kick to 30% in the overhand throw) except for the catch, where the percentage of explorer-level children remained at 4% after the intervention program. In the control group (left side of the figure), there was also a decrease in the percentage of children classified at the explorer level after the intervention program in dribble (from 18% to 16%) and the overhand throw (from 58% to 44%). However, there was an increase in the catch (from 5% to 8%). The underhand throw (from 37% to 47%) and no change in the kick (13%). For this analysis, children from the control group with one and two classes per week were combined into one group, and children from the intervention group with one and two times per week were combined into another group.

A similar trend is evident in locomotor skills (Figure 6). In the experimental group (right side of the figure), there was also a decrease in the percentage of children classified at the explorer level in all tasks after the intervention program: slide (from 3.8% to 2.0%), single leg (from 9.0% to 2.0%), gallop (from 38.0% to 15.0%), hop (from 34.0% to 4.0%), horizontal jump (from 25.0% to 2.0%) and leap (from 15.0% to 4.0%). In the control group (left side of the Figure), there was also a decrease in the percentage of children classified at the explorer level after the intervention program in slide (from 2.6% to 0%), in single leg (from 8.0% to 3.0%), in gallop (from 29.0% to 26.0%) and in horizontal jump (from 16.0% to 10.0%). However, the number of explorer children increased in the hop (from 21.0% to 24.0%) and leap (from 8.0% to 13.0%) tasks.

## 4. Discussion

This study showed that a new PE method of teaching FMS was significantly better than the traditional/official one in developing children’s FMS. There was evidence of more positive individual differences in the responses to the new method than in the control group. Furthermore, the trainability was relatively similar in both groups, given the spread of the distribution of individual changes (see Figure 3). Finally, there was evidence of greater moves from explorer-level children to higher proficiency FMS levels (adventurous and wizard) than observed in the control group. This difference can be explained by the new method’s structural approach as well as the classes’ didactical strategies. Although Portugal’s national program includes the development of FMS in its proposal, it does not present a set of conditions that could enhance the development of these skills. For example, in our method, we emphasize the importance of the structure of sequential lessons that allow children to experience the same skill in different conditions (diversity) and gradually add new components to the practice, making it increasingly complex, enabling children to have adequate time and conditions to refine their movement patterns [49]. Recent research has identified that this condition is more favorable to the development of FMS than physical education classes that lack this structure. Specifically, those classes work on different skills in each session without considering the complexity level of the task [50].

Regarding the complexity level, we must emphasize that our new method guides teachers to respect individual differences (student constraints) and establish different difficulty levels within the same task during the class. For example, in a game where the goal is to hit the ball into a basket, the teacher is guided to indicate different basket options, some higher, some lower, larger, or smaller. Research indicates that classroom environments that encourage students to actively participate in decision-making, such as choosing tasks or goals, stimulate autonomy in learning and motivate students to participate in physical education classes [51,52].

Targeted instruction, particularly designed for children grappling with more learning difficulties, can potentially generate a significant impact [53,54]. One of the first studies to investigate the effects of instruction on FMS performance demonstrates that practice without instruction may not be sufficient to improve movement patterns [55]. To address this, our method was also designed to aid teachers in providing tips on specific components of each FMS. In summary, although the ‘traditional’ classes applied to the control group aimed to develop FMS, they did not follow any of the mentioned conditions. This leads us to infer that the rules established in our method favored the development of FMS.

Our second aim was to estimate children’s trainability, i.e., their interindividual differences in response to the new method beyond random variation. Previous research with interventions aiming at children’s FMS development [19,20,21,22] never addressed the issue of heterogeneity in individual responses. For example, the Logan, et al. [56] meta-analysis on the effectiveness of motor skills interventions in children and the Van Capelle, et al. [57] systematic review and meta-analysis on interventions to improve children´s FMS, although indicating a substantial amount of heterogeneity among studies´ effectiveness, did not tackle the theme of individual variation in responses within any of these interventions. The seminal report from Bouchard and Rankinen [47] on individual responses to aerobic exercise training is important for motor development researchers, especially those dealing with changing children’s FMS proficiency levels within the school setting. In the present article, we showed (see Figure 3) that children vary substantially in their FMS changes in object control, locomotion, and motor competence. Based on the Newell [42] constraints model, this may be due to individual and task constraints, namely children’s previous motor experiences, physical growth and body composition differences, psychomotor readiness and/or motivation, and the intrinsic characteristics of each skill. This is true for both the control and experimental groups. Yet, children from the experimental group showed, systematically, more positive changes than the control group. This issue is linked to trainability or the positive response beyond measurement and sampling errors.

There is a methodological debate on how best to identify true from false individual differences in the physiological response to interventions in adults [27,58,59] as well as a refutation to the idea of non-response to exercise training [29,60,61]. In our paper, we relied on Hopkins [28] and Dankel and Loenneke [48] suggestions to address these issues, given that children are not expected to respond similarly. If this were to happen, then the variance of changes would be zero, which was not the case in the control or experimental groups. Unfortunately, we could not locate a paper or a book chapter dedicated to these topics in FMS development, especially in interventions within the school setting, which poses difficulties in comparing our results. In any case, we identified differences, but the high and low responders were very few, which may be due to the duration of the study. Another important caveat in this regard is the following—irrespective of the statistical method used to classify responders, there is no indication in the new method, nor the traditional/official program, about a valid performance standard statistically derived or substantively suggested to help the PE teacher and researchers.

Despite this problem, we believe that in the school context, beyond the relative importance of identifying putative high responders to PE classes during the seasonal periods or across annual planning, the PE teacher is expected to be more concerned with children less proficient in their FMS levels and find remedial plans to solve their difficulties. Yet, the PE teacher does not know, unless she/he systematically assesses the child, which parameters to consider in FMS performance to help her/him define a low responder relative to her/his peers within the class. The lack of these performance standards is still a serious challenge for PE teachers’ assessments and interpretation. Without sound criteria, it is very hard to tailor individual interventions or track progress [62]. Future research should establish evidence-based FMS performance benchmarks akin to a minimally important difference considering children’s characteristics. This can significantly improve the quality of PE programs and promote children’s holistic development. It would be particularly beneficial for children who are currently less proficient in their FMS, providing them with the support and guidance they need to improve. Our results suggest that the new method could effectively enhance motor competence in children at various developmental stages, helping students overcome motor proficiency barriers [37,63]. Additionally, because it is flexible and adaptable to various curricula frameworks (e.g., Brazil’s National Common Curricular Base, BNCC), the new method integrates the development of critical, cultural, social, and historical domains, addressing both motor development and cultural knowledge. Finally, it also provides PE teachers with a solid theoretical foundation, enabling them to justify-content selection for different age groups based on established research.

Notwithstanding the importance of this study, at least two limitations must be recognized. The first relates to the sample size, which may hinder statistical power in detecting real changes beyond sampling and measurement errors. Studies conducted by Zask, Barnett, Rose, Brooks, Molyneux, Hughes, Adams and Salmon [19], Bryant, Duncan, Birch and James [21], and Roach and Keats [20] related to children’s FMS changes in the school context used samples equivalent to or even smaller than the one used in the present article and a similar tendency was evident in adult physiology research, as reported by Davidsen, et al. [63], Vikmoen, et al. [64], Tardif, et al. [65], and Enes, et al. [66]. Irrespective of this trend, it is important to note that our design’s post hoc power analysis revealed values ranging from 0.70 to 0.90. This way, we are confident in our results and conclusions. The second limitation concerns the original design, i.e., the control group was not a “classical” control group. This group also underwent a “treatment,” which was their traditional/official PE classes, and a similar situation was reported by Bryant, Duncan, Birch and James [21], as well as in the systematic reviews and meta-analysis done by Logan, Robinson, Wilson and Lucas [56] and Van Capelle, Broderick, van Doorn, Ward and Parmenter [57]. Given that children in this group also responded differently to their traditional PE classes, we could not identify consistent differences in FMS trainability between both groups. It is also important to note that although the study was done in an ecological setting, no problems were reported by children and/or PE teachers working in both groups (control and experimental). Irrespective of these limitations, the present study also has strong points. The first is that it was conducted in the school setting, linked to ecological validity [21]. The second relates to using a new technological device to assess children’s FMS, i.e., the Meu Educativo^®^ app. The third is that we used twelve FMS, which is somewhat rare in recent research in the school setting. Even though the assessment of FMS in children is prone to errors, our assessments were highly reliable. Fourth, we used different statistical techniques to best capture children’s individual differences in response to the intervention.

## 5. Conclusions

The new method for developing children’s FMS proved significantly more effective than the traditional/official one. While the longstanding traditional/official program has its merits, it lacks explicit emphasis on FMS development—a crucial component during a child’s primary school years. On the other hand, the new method strongly emphasizes enhancing motor skills while simultaneously nurturing cultural awareness and critical thinking abilities. These elements are instrumental in a child’s physical and motor development, potentially impacting their ability to engage and maintain an active and healthy lifestyle. However, it is important to note that individual responses to this new method varied considerably, highlighting the need for personalized teaching strategies. This observation points to the complex nature of child development and the importance of adaptable educational approaches catering to children’s diverse needs and learning styles.

Notwithstanding the relevance of the study findings, future research should consider at least three important avenues: (i) extend the method to the entire academic year, whatever the number of physical education classes available for children (one or two), and investigate its effects not only in fundamental movement skills development but also in children’s perceived motor competence; (ii) investigate how these changes will increase children’s sports participation and proficiency in combining motor skills, especially those related to sports; (iii) follow their motor development and link its sustainability in terms of showing more active and healthy lifestyles.

## Figures and Tables

**Table 1:** Mixed-model results (regression coefficients, *β*, and their standard errors (SE), z, *p*-values, and 95% confidence intervals (95% CI) for object control and locomotor skills as well as motor competence (statistically significant results are in bold).

Variables	*β* ± SE	z	*p*-Value	95% CI
**Object control skills**				
Intercept	3.65 ± 2.07	1.97	0.078	−0.41 to 7.71
Age	0.78 ± 0.29	2.68	**0.007**	0.21 to 1.35
Sex	2.69 ± 0.30	8.91	**<0.001**	2.09 to 3.28
Condition	0.22 ± 0.86	0.26	0.796	−1.47 to 1.92
Time	0.11 ± 0.43	0.24	0.808	−0.74 to 0.95
Condition-by-Time	1.13 ± 0.57	1.97	**0.048**	0.008 to 2.24
**Locomotor skills**				
Intercept	11.72 ± 1.95	3.58	**<0.001**	1.06 to 3.63
Age	0.48 ± 0.28	1.70	0.089	−0.07 to 1.03
Sex	0.06 ± 0.33	0.18	0.859	−0.59 to 0.71
Condition	−0.26 ± 0.48	−0.55	0.584	−1.19 to 0.67
Time	0.08 ± 0.50	0.16	0.874	−0.89 to 1.05
Condition-by-Time	2.34 ± 0.65	3.58	**<0.001**	1.06 to 3.63
**Motor Competence**				
Intercept	14.37 ± 3.55	4.05	**<0.001**	7.41 to 21.32
Age	1.40 ± 0.51	2.75	**0.006**	0.40 to 2.41
Sex	2.72 ± 0.55	4.99	**<0.001**	1.65 to 3.79
Condition	0.06 ± 1.11	0.05	0.86	−2.11 to 2.22
Time	0.18 ± 0.79	0.23	0.816	−1.4 to 1.74
Condition-by-Time	3.50 ± 1.04	3.32	**0.001**	1.42 to 5.52

**Table 2:** Adjusted means (M) ± standard-errors (SE) at baseline and end of the study in the control and experimental groups.

Variables	Control Group		Experimental Group	
	Baseline	End of Study	Baseline	End of Study
	M ± SE	M ± SE	M ± SE	M ± SE
Object control skills	9.8 ± 0.7	10.1 ± 0.6	9.9 ± 0.7	11.3 ± 0.6
Locomotor skills	14.9 ± 0.4	14.6 ± 0.3	15.0 ± 0.4	17.0 ± 0.3
Motor competence	24.7 ± 0.8	24.7 ± 0.7	24.9 ± 0.8	28.4 ± 0.7

## Data Availability

Data will be available within reasonable requests within the rules of the University of Porto, Porto, Portugal data sharing.
